# Balancing social and economic factors - explorative qualitative analysis of working conditions of supervisors in German social firms

**DOI:** 10.1186/s12995-021-00342-y

**Published:** 2022-01-25

**Authors:** Ann-Christin Kordsmeyer, Ilona Efimov, Julia Christine Lengen, Annegret Flothow, Albert Nienhaus, Volker Harth, Stefanie Mache

**Affiliations:** 1 Institute for Occupational and Maritime Medicine (ZfAM), University Medical Centre Hamburg-Eppendorf (UKE), Hamburg, Germany; 2 Department of Nutrition and Home Economics, Hamburg University of Applied Sciences (HAW Hamburg), Hamburg, Germany; 3 Department for Occupational Medicine, Hazardous Substances and Health Sciences (AGG), German Social Accident Insurance for the Health and Welfare Services (BGW), Hamburg, Germany; 4 Competence Centre for Epidemiology and Health Services Research for Healthcare Professionals (CVcare), Institute for Health Services Research in Dermatology and Nursing (IVDP), University Medical Centre Hamburg-Eppendorf (UKE), Hamburg, Germany

**Keywords:** Job demands, Job resources, Leadership, Occupational health, Social enterprises, Social firms, Working conditions, Supervisors

## Abstract

**Background:**

Social firms are companies on the general labour market which provide employment to people with severe disabilities. In this setting different job resources are offered for its employees, including social support or flexibility in terms of working hours, tasks or pace of work. However, to date, only limited evidence exists on the work and health situation of supervisors in social firms. Therefore, the study aims to explore job demands and resources of supervisors in social firms to increase knowledge in a little researched field and to develop recommendations for action on workplace health promotion.

**Methods:**

Sixteen semi-structured telephone interviews were conducted with supervisors of social firms in the North of Germany within July and November 2020. Different sectors such as gastronomy or cleaning services as well as employment opportunities for people with different types of disabilities were included. The interviews were recorded, transcribed verbatim and analysed following the qualitative content analysis according to Mayring (deductive-inductive approach).

**Results:**

Overall, a heterogeneous composition was aimed for in terms of age and gender distribution (68.75% were male and between 32 and 60 years old). Supervisors reported various job demands in social firms, including for instance emotional demands, conflicts between social and economic objectives, conflict management, exposure to heat, heavy lifting or constant standing. In contrast, a high meaning of work, possibilities in shaping the structure of the social firm, social support of colleagues or the management and the provision of an ergonomic work environment were highlighted as job resources. Further person-related demands (e.g. own expectations) and resources (e.g. patience) were underlined as well.

**Conclusion:**

First exploratory insights were provided with reference to job demands and resources for supervisors in social firms. The overview on working conditions underlines the significance of a supportive work environment taking structural and behavioural-related implications into account to reduce demands and strengthen resources. Further interventional research is needed regarding the development, testing and evaluation of workplace health promotion interventions in social firms.

**Supplementary Information:**

The online version contains supplementary material available at 10.1186/s12995-021-00342-y.

## Background

Gaining and maintaining employment results in several benefits for people with disabilities, like the development of self-esteem, social skills, autonomy or the feeling of belonging to the community [1]. One approach enabling people with disabilities to hold employment is to maintain businesses with supportive work environments such as social firms [e.g. 2, 3]. Social firms – a type of social enterprise – are characterised by several prominent factors, which differ from other options to gain employment like sheltered workshops or other programs on the general labour market designed for people with disabilities (see definition by Corbière et al. [4]). As a result of country-specific differences or management approaches, social firms are also known as “affirmative businesses, adapted enterprises, cooperatives, collectives [or] consumer/survivor-run businesses” ([4], p. 39).

In Germany, social firms must employ at least 30% and can employ up to a maximum of 50% of people with severe mental, intellectual, physical, sensory or multiple disabilities (§ 215, Book Nine of the German Social Code (SGB IX)). In 2019, there were 965 social firms in Germany employing about 13,550 severely disabled people with a significant proportion of employees with mental disabilities (28%) followed by those with intellectual disabilities (24%) [5]. Overall, social firms are mainly located in the areas of gastronomy (18%), industrial or craft services (13.4% and 12.6% respectively), retail (12.6%) gardening and landscaping (11.4%), or facility management (11.2%) [6].

In social firms, different types of job resources were provided for employees [7], like social support [2–4, 8–27], flexibility regarding work schedules, work tasks or pace of work [2–4, 8, 10–13, 16, 19, 22–24, 26], structured work tasks [2, 3, 8, 10–13, 19–23, 25–27], different options for training like the gradually introduction of tasks or access to educational resources [4, 11, 16, 19, 25] and increased levels of job security [3, 8, 10–12, 19, 23, 24, 26, 27]. One essential resource is personified by supervisors themselves promoting a supportive work environment [2, 3, 8, 11, 19, 26]. Within the framework of a workplace health management, leadership including the management as well as direct supervisors incorporates a key role, since they influence the work situation of their employees in their everyday lives in different ways [28]. Adequate communication structures, respect and appreciation, patience in teaching skills, mutual understanding, the absence of conflicts, pressure or demands were described as contributing to the quality of work life of employees in social firms as well as a sense of humour, enthusiasm, expertise, the provision of support and to know the employee’s needs [2, 11].

However, there is less evidence on working conditions for supervisors compared to those for employees with disabilities. In the current state of research, several job demands are reported for supervisors based on the organisational structure of social firms. Especially supervisors face “a double challenge” in managing both economic and social objectives in social firms ([13], p. 67). Although employee participation should be included in the workplace culture and leadership style, it was frequently impeded by business realities, missing awareness of supervisors, lacking feedback for employees or involvement in decision-making processes [13]. Being able to work with employees with disabilities, Corbière et al. [4] reported that between 47.1 and 76.9% of the participating supervisors received training about mental health conditions. Additional requirements are also underlined by the results from research on leadership for employees with autism spectrum disorders who have multifaceted preferences on leadership resulting in different leadership behaviours instead of one main leadership concept [29].

All in all, it is necessary to gain insight into working conditions of supervisors in addition to employees with disabilities not only to strengthen their health and productivity-related outcomes in the first place, but also to provide enough resources to support their employees and respond to their needs. If supervisors are exposed to considerable pressure or demands, fewer resources are available for supporting their employees. Last but not least, they also serve as role models, on the one hand with regard to work performance and attitudes, and on the other hand regarding their own health [28].

### Theoretical framework

To examine working conditions of supervisors in German social firms the Job Demands-Resources model (JD-R model) developed by Demerouti and Bakker [30–32] was chosen as a theoretical framework of the study. The model follows the assumption that various professions face particular health relevant factors linked to job stress, taking two main categories into account: job demands and job resources. Job demands describe e.g. social, physical or organisational occupational characteristics like time pressure or challenging interactions with customers which call for ongoing endeavour or coping capabilities. According to the German DIN EN ISO 10075 [33] they are understood as neutral impacted by external factors, which can have both negative and positive effects on employees’ health depending on the individual reaction to job demands. On the contrary, job resources apply to characteristics of the job which stimulate achieving work-related goals, diminish job demands and its physiological and psychological consequences and promote individual growth and development. The model can be transferred to different occupational settings regardless of the encountered job demands or resources [30–32].

To categorise job demands and resources further, relevant work characteristics according to the Joint German Occupational Safety and Health Strategy (GDA) were used, including work tasks, work organisation, social relations and work environment. Those work characteristics were developed as a result of a systematic processing and evaluation of available scientific knowledge important for the assessment of mental health at work by the German Federal Institute for Occupational Safety and Health (BAuA) [34]. Additionally, personal demands and resources are examined as well, like social or problem-solving competencies or attitudes [35, 36].

### Study aim and research questions

Since 2018, German social firms are obliged to implement measures for workplace health promotion (§ 216, Book Nine of the German Social Code (SGB IX)), which depicts a significant difference to other companies in the general labour market. However, currently, there is no specific research available examining the work situation of supervisors of social firms in Germany. Therefore, this study aims to gain exploratory insights into job demands and resources according to the criteria of the GDA [34], wherefore two main research questions are addressed.

1. What job demands and resources were reported for supervisors in social firms in terms of their work tasks, organisation, social relations or work environment?
2. What personal demands and resources were examined for supervisors in social firms?

Thus, a basis for the development of target-group specific implications for workplace health promotion could be deduced.

## Methods

### Study design

To gain insight into job demands and resources of supervisors in social firms 16 semi-structured interviews were conducted between July and November 2020. The qualitative study approach was chosen to gain initial insight into the topic in this specific occupational setting [37]. Prior to the interviews, participatory observations were conducted in participating social firms in the North of Germany, wherefore initial trust could be established. No personal relationship existed with any participant in advance.

### Participant selection

Participants were selected via convenience sampling. After the initial contact through the participating observations, a request was made to the social firm’s management as gatekeeper. Inclusion criteria for the participating supervisors were defined beforehand including their age (at least 18 years old), working hours (at least 18 h per week), language (German), position (supervisor in a social firm according to § 215 ff., Book Nine of the German Social Code (SGB IX)), professional experience (at least half a year) and their work tasks (direct contact to employees with disabilities). Within the selection process two persons ignored the invitation to participate within the interviews because of time pressure continuing business operations or due to the COVID-19 pandemic.

### Data collection

The interviews were conducted by the first author of the study, a female health scientist (M.A.) employed as research associate. She was experienced in conducting qualitative interviews. Telephone interviews were chosen to facilitate flexibility and practicability during the COVID-19 pandemic. The data was collected when supervisors were at their workplace or at home. Participants were encouraged to seek a quiet place like their office when conducting the interview without being disturbed. Nevertheless, one interview participant was interrupted by colleagues during the interview. Before the interviews, participants gave informed consent after receiving information about the study objectives, methods, analysis and data protection regulations. Overall, participation in the study was voluntary. A semi-structured interview guide was developed according to Witzels’ problem-centred interview technique (PCI [38]) to evaluate on experiences, perceptions and reflections of the interviewee on a specific problem. The guide was developed referring to the displayed research questions and the theoretical framework of the study (JD-R model). Various main questions, detailing questions and control questions were included based on the SPSS principle (collect, check, sort, subsume) according to Helfferich [39]. At the end of the interviews, socio-demographic data (like gender or age) was collected and postscripts were used, including conversational content, situational and non-verbal aspects, thematic emphases and interpretation ideas [38]. The interview guide was tested beforehand to receive feedback from colleagues, to check the duration of the interview and the comprehensibility of the questions. The topics job demands (e.g. perceived challenges at work) and job resources (e.g. perceived positive, supportive characteristics at work) were included in the interview guide.

The interviews were conducted in German, were audio recorded and lasted on average 46:19 min (range: 00:30:19–01:11:00). Repeat interviews were not conducted as this may affect the relationship between researcher and interviewee and in turn the data collected [37]. Likewise, transcripts were not returned to interviewees for comment or correction purpose and feedback was not collected from participants.

### Data analysis and reporting

The audio recordings were transcribed according to the rules of Kuckartz [40] and anonymised in the same course. The transcripts were checked by the first author (A.C.K) and encoded using the software MAXQDA Analytics Pro (version 12, VERBI GmbH, 2016), that made it possible to manage text documents, define main and sub-categories, create a category system, assign text passages to categories, and create a synopsis of the coded text segments [41]. After encoding the first interview, results were discussed by two researchers (A.C.K. and J.C.L.) and the defined sub-categories and allocation of text segments were compared until a consensus was reached. The following interviews were encoded by the first author herself (A.C.K.). In case of upcoming uncertainties on text passages or codes, discussions in regular project meetings took place. To ensure a systematic and transparent data analysis, a deductive-inductive approach was chosen referring to Mayring’s qualitative content analysis (controlled analysis of fixed communication guided by content analytical rules and categories following a stepwise approaches, without quantifying results [42]). Main categories were created in a deductive way referring to the theoretical framework of the study (job demands and resources) and allocated to the categories of work tasks (e.g. completeness of tasks, work autonomy, responsibilities or emotional demands), work organisation (e.g. working time, workflow or communication and cooperation activities), social relations (related to colleagues or supervisors/management) and work environment (e.g. physical and chemical factors, the workplace design and work equipment) each based on the criteria of the GDA [34] as well as personal ones [35, 36]. Further sub-categories of the data were established in an inductive way [42]. For illustrating results, quotes of participants were translated to English. The COREQ-Checklist (Consolidated criteria for reporting qualitative research [37]) served as a basis when reporting the results of the study (displayed in the supplementary material).

### Ethical considerations

The study was approved by the Ethics Committee of the University Medical Centre Hamburg-Eppendorf (UKE), Germany (LPEK-0051).

## Results

### Characteristics of the study population

Table 1 presents characteristics of the 16 participants working in five different social firms. Supervisors were engaged in food and beverage service activities, services to buildings and landscape activities, printing and reproduction of recorded media, office administrative, office support and other business support activities or wholesale of bicycles and their parts and supplies (based on [43]).

**Table 1:** Participant characteristics of supervisors in social firms (*n* = 16)

Variable	n	%
*Gender*		
Male	11	68.75
Female	5	31.25
*Age*		
30–40 years	7	43.75
41–50 years	3	18.75
51–60 years	6	37.50
	Mean: 45.7 years, Range: 32–60 years	
*Working experience in current social firm*		
Less than a year	1	6.25
1–3 years	9	56.25
More than 3 years	6	37.50
	Mean: 5.0 years, Range: 8 months–20 years	
*Weekly working hours*		
Less than 30	1	6.25
30–34	2	12.50
35–40	13	81.25
	Mean: 37.4 h, Range: 18–40 h	
*Subordinate employees*		
1–5	2	12.50
6–10	7	43.75
11–15	4	25.00
15–20	2	12.50
More than 20	1	6.25
	Mean: 11.3 employees, Range: 5–25 employees	

### Job demands

Referring to the proposed theoretical framework, Table 2 includes an overview of sub-categories referring to job demands and resources as well as personal ones of supervisors in social firms.

**Table 2:** Overview of job and personal demands and resources of employees in social firms

	Demands	Resources
Work tasks	• Guiding work tasks • Emotional demands • Customer contact	• Meaning of work • Variety of work tasks • Work autonomy • Pleasure in working with people
Work organisation	• Conflicts between social and economic objectives of the company • Work absences of employees • Collaboration with employees • High work intensity and time pressure • Working hours • Undefined communication structures • Accessibility and dissolution of boundaries • Low financial remuneration • Long daily commute • Lack of initial training • Negotiations with the integration office	• Participation in shaping the structure of the social firm • Trainings and seminars • Meetings on a regular basis • Substitution arrangements • Cooperation and exchange within the social firm • Cooperation and networking with external organisations or players • Working hours
Social relations	• Lack of social support • Conflicts management for employees • Conflict management with supervisors involved • Communication with hearing impaired employees • Lack of appreciation	• Working atmosphere • Social support of colleagues • Social support of the management • Pedagogical support • Appreciation • Customer satisfaction • Company outings • Provision of a social psychiatric service
Work environment	• Heat • Limited shower facilities and small changing rooms • Work equipment placed too high • Heavy lifting • Constant standing • Technical problems	• Provision of an ergonomic work environment • Spacious work environment • Use of work equipment from the parent company
Personal	• Learned behavioural patterns in the career • Own expectations and emotions • Wanting to help	• Patience • Empathy • Optimism • Dealing with perfectionism

#### Category work tasks

##### Guiding work tasks

Guiding work tasks for employees were described as challenging, e.g. when employees were less able to think or work in advance, when making unpleasant decisions or when the supervisor’s expertise was needed in many different places. Overall, supervisors tried to provide the employees enriched working conditions with new learning experiences and a variety work tasks.

*“Then this, yes thinking in advance (...) is just not present here, you have to tell them ‘okay, do this, do this, do this, look there again’ (…), it is just the whole thing around, to really look if everything is ready, if everything is cleaned, if everything has been done, to work in advance, yes that is just the difficulty, the difficulty of it, that it is sometimes very complex”. (Supervisor #14, male)*

Other supervisors described difficulties in learning to delegate work tasks to employees.

*“Because that's our job, we're supposed to delegate, we're supposed to make sure they do it right, we're supposed to teach them how to do it, we're supposed to train them, (…) we're supposed to be able to sort out small problems and so on and that, we don't have the time to do everything by ourselves.” (Supervisor #4, female)*

Furthermore, tasks in addition to the actual work activity of the supervisor were described, including for instance auditing or administration tasks, as well as monthly settlement, waste disposal or dealing with craftsmen.

*“So I have all kinds of things on my mind here all day long. So that's the (...) production, but people also come to me when something is wrong with the disposal.” (Supervisor #14, male)*

##### Emotional demands

Job demands referring to employee’s sensitivities were also mentioned. In spite of the consideration of individual needs in planning processes, employees were sometimes described as unable to accept the support options provided by supervisors.

*“Well, there are also sensitivities of people that you have to respond to, or when people feel stressed and you have to see how to deal with it, you have to take it seriously, you can't just walk away from it, it's your own perception, even if it may not be right, it's there in any case, and that takes energy.” (Supervisor #5, male)*

Since employee’s fears and concerns were described as more present, supervisors also reported emotional demands and worries about their employees, e.g. when they do not show up at work or due to their private problems.

*“Yes, so you also get personal backgrounds sometimes, which are quite tragic, there are also employees who perhaps also share a lot of personal things about themselves and sometimes it's already a bit too much for me, I sometimes take that with me, but it keeps within limits.” (Supervisor #3, male)*

Overall, a high number of conversations during a work day were reported.

*“Also so the amount of people can be very challenging here, right, when many people want something from you.” (Supervisor #3, male)*

##### Customer contact

Supervisors described challenges during contact with customers, e.g. when they requested a lot of appreciation, couldn’t make one’s choice or when customer contact could only be taken over by the supervisor.

*“Customer contact, yes, can be a challenge, sure, so definitely, (...) the people can definitely be exhausting, they also demand a lot of appreciation, so I've already had to hear that I didn't raise my people well, they never greet (...) so you have to be careful, so I always say hello to them and be polite.” (Supervisor #5, male)*

#### Category work organisation

##### Conflicts between social and economic objectives of the company

Another topic which was added included conflicts between economic and social objectives. Those conflicts often resulted from a limited performance of the employees in combination with high customer expectations regarding speed, implementation and price policies. Other demands resulted due to additional socio-pedagogical activities such as personal conversations or when supervisors had to deal with acute challenges of employees during high workloads (e.g. when conflicts aroused during high order volumes in the gastronomy sector).

*“And because I also carry out these socio-pedagogical activities, i.e. personal, emotional discussions with the people, when there is again somehow a strained atmosphere in somebody’s home or something is wrong. If I notice that the boys are strained, then I have to sit down together for half an hour, drink a coffee, eat a piece of cake and then they should have the space to open up a bit and tell again what's going on. Of course, if I do something like that, I can't do anything else at the same time, because, as I said, my attention should be focused on the person. If I then write an e-mail on the side, then I can just as well refrain from it, that doesn't get anyone anywhere.” (Supervisor #11, male)*

*“I'm the economic factor between the, the employee or staff or something. That means I have to juggle in between again and again, and sometimes that's quite a challenge. When I think. ‘Man, that's actually not so bad. This is going to take ten minutes. Why have you been at this for two hours?’ But I can't say that like that. I have to then somehow see that I can somehow manage it. So that, that's exactly the point, that's good as well as bad.” (Supervisor #14, male)*

Therefore, supervisors tried to focus more on employees’ resources than on economic results or refrain from orders that are not solvable for employees, because they want to maintain personnel resources in a long-term and sustainable way without putting the employees at risk.

##### Work absences of employees

A topic which is connected with the above mentioned conflicts was work absences of employees due to mental health conditions which must be intercepted or represented by the supervisor, for instance by means of overtime or reorganisation (especially a challenge in case of high workloads or deadlines for customer orders).

*“But it is challenging (...) when someone drops out at short notice or is emotionally unable at the moment and then simply drops out in the middle of it or just can't perform as they otherwise would, I think that's a challenge.” (Supervisor #13, female)*

In general, staff scheduling was described as challenging, when considerations for absences or emotional crises had to be taken into account.

##### Collaboration with employees

Challenges in collaboration with disabled employees were added as a job demand e.g. due to the forgetfulness of the employees or when the same questions were often asked (also with employees holding outsourced jobs of sheltered workshops). This was especially challenging in situations with a high work intensity.

*“If the employees don't listen and then keep coming back, it's fun to teach them again, but sometimes just in stressful situations, you think, ‘Oh, I've already explained that to you five times,’ so I don't say that, but I think to myself, ‘How many times do I have to explain that to you,’ because you like doing it, but sometimes it's stressful.” (Supervisor #4, female)*

##### High work intensity and time pressure

Moreover, a high work intensity was described by supervisors, e.g. when many conversations were conducted or there was a high workload in the kitchen as applied for the gastronomy sector, wherefore work time was described as not sufficient.

*“So of course there are stressful days, today for example was a very stressful day, because today was incredibly busy, (...) like in a normal kitchen, (...) just with more people, it is then also already stressful.” (Supervisor #10, male)*

An additional unpredictability was also stated when working in the gastronomy sector.

*“You can never plan gastronomy, it's always a surprise every day what happens, yes.” (Supervisor #10, male)*

Supervisors added that they worked under time pressure due to deadlines or when something was forgotten.

*“So the challenge is generally always the time, that you are often limited in time (…), that there are always deadlines that you have to meet and so, that is such a challenge.” (Supervisor #15, male)*

##### Working hours

Therefore, supervisors informed about high numbers of overtime hours, even despite compensation possibilities. As a result, e.g. only little time for doing sports was left.

*“Yes, insofar as I still have to carry around a hundred hours of overtime in a forty-hour week, even though I'm always at home for a day. So overtime is simply the issue there.” (Supervisor #14, male)*

Moreover, challenges in organising working time were added, e.g. when working time extends over the day or when organising working hours at weekends and during lunch times, respectively.

##### Undefined communication structures

Additionally, supervisors underlined that the social firm’s structures still needed to be defined, e.g. referring to lacking communication strategies and means, work processes, substitution arrangements or the handling of customer inquiries.

*“Yes, it's quite different, so there's actually a lot of communication here, but sometimes something falls through the cracks or it's a last-minute agreement, I think there's just a lack of fixed communication strategies or means of communication or work, i.e. fixed work processes, even in the case of a substitute or so that's sometimes a bit difficult.” (Supervisor #13, female)*

##### Accessibility and dissolution of boundaries

Another topic of work organisation was the supervisor’s permanent accessibility, also in the context of work requests on the weekend in messenger groups.

*“What I really feel burdened about is actually that I am simply always reachable. That is stressful, that is of course also the case when I am on vacation, then I don't turn off my cell phone, because I use my work cell phone at the same time as my private cell phone. (...) and now it's also the case that I get a lot more calls spread throughout the day and sometimes the calls, as I said earlier, come at the most inconvenient times, when I'm doing something recreationally with my friends or doing sports, and now it's the case that even when I'm on vacation, I get calls all the time and I still forward them and can't quite tear myself away from work.” (Supervisor #6, male)*

Other topics from the interviews referred to a low financial remuneration (wherefore individual supervisors worked additional part-time to earn more money), challenges due to the daily commute or a lack of initial training for the supervisors. If in smaller social firms the direct supervisor also held the position of the managing director, challenges in negotiations with the integration office (a German authority who is responsible for funding construction, expansion, modernization and equipment including business consulting and other special efforts) were also mentioned as demanding.

#### Category social relations

##### Lack of social support

For supervisors, a lack of social support was mentioned as a job demand, especially when they worked alone in the department without a disability.

*“So it's really challenging and energy-consuming that I'm alone, I don't have anyone with me in the company who is, let's say, without a disability or who can also take the reins properly and everything.” (Supervisor #7, female)*

*“Of course, so I am now just looking for supportive manpower that we here still/ So also a fully resilient person who also again so a bit an assistance of my, for my, occurs for me as an assistant (…) so a few of the processes that I'm doing here at the moment can also be done while I'm on vacation and yes, can also work off a bit again, if worst comes to worst and yes. Then I just, then I just look for appropriate support. (...) That is (…) not an unknown phenomenon that care ratios in such facilities are then also difficult to produce to the extent that one would actually need them.” (Supervisor #11, male)*

In the same way, supporting arrangements weren’t available for certain tasks, e.g., when customer contact must be handled by supervisors (as a single point of contact).

##### Conflict management for employees

Next, support in case of conflicts among employees were mentioned as burdensome, which aroused due to different skills of the employees or with non-target group employees.

*“The most problems cause employees who do not come from the target group. They don't quite understand and then you have to exchange them from time to time, right? Because then they bring in unrest and that's not so good, of course.” (Supervisor #16, male)*

Therefore, supervisors had to reconstruct, offer different tasks or dismiss certain employees.

##### Conflict management with supervisors involved

In the same vein, conflicts were also mentioned in which supervisors themselves were involved. On the one hand, when perceiving power relationships in a different way despite flat hierarchies or when supervisors were played off against each other.

*“At the moment, you rather have the feeling that (…) others are getting a bit more of the reins in their hands, that they sometimes that you have the feeling that they feel like something better in the meantime, although we are all still equal and a lot of these things (…). And untruths are also constantly being spread to some extent, that's just this problem.” (Supervisor #7, female)*

On the other hand, conflicts between supervisors and employees were added, e.g. due to high levels of employee’s aggressiveness or when supervisors were newly introduced to a team.

*“So clearly I have an employee who likes to get very, very aggressive sometimes and then I actually have him under control, but despite all that, it then just has to be discussed, right?” (Supervisor #16, male)*

As an additional job demand, conflicts between the supervisor and the management were depicted, e.g. due to changes in personnel, a different professional socialization and resulting views on staff management (technical-commercial vs. socio-pedagogical approaches), lacking communication between management and supervisors or denied requests for support.

*“Keyword differences with the management, on this flat management level, so that has also not (...) necessarily had positive effects on my psyche.” (Supervisor #9, male)*

##### Communication with hearing impaired employees

Supervisors described challenges, when communicating with hearing impaired employees, e.g. when dealing with urgent customer complaints or when supervisors wanted to involve hearing impaired colleagues more in team interactions.

*“Then communication with hearing impaired people is always a challenge. We manage it relatively well and there is always a pen and a piece of paper available. But I think it is very challenging for both sides, because hearing impaired people understand the world differently and see it differently than we hearing people do. Yes, both sides have to go further and further and more and more towards each other, always try to find more understanding for each other, why a hearing impaired person reacts the way he does, we hearing people just don't understand, because they simply have a different way of dealing with each other”. (Supervisor #3, male)*

##### Lack of appreciation

Both a lack of appreciation from the management as well as from customers were presented as job demands by supervisors.

*“So I've been doing this for a few years now (...) people are always stingy with appreciation, it's like that, I've learned that if you don't say anything, that's always good, because if something is bad, then the guests say something or our manager says something, of course he should, I ask, I ask for feedback, but then also say something positive, would also be good for them, not for me, but for the guys in the kitchen, they are happy about it, if it doesn't come from me, but from the manager or who knows who was there”. (Supervisor #10, male)*

Other examples were given, when only little appreciation from the management was provided despite a variety of support services offered to employees (support with debts, phone calls, plans, photos for support).

#### Category work environment

In terms of the work environment, exposure of the supervisor to heat was mentioned as demanding, as well as limited shower facilities and small cramped changing rooms.

*“External, external conditions I have noticed, (…) when it was very hot these days. Then I, I noticed that I'm no longer capable, so there was at 4 p.m., I was already out of breath, I just yawned, so that's my, that's my personal story, yes, I was totally exhausted.” (Supervisor #9, male)*

Furthermore, kitchen equipment was placed too high, supervisors had to lift heavy things when no support was available and perform activities that require constant standing.

*“If I have to lift something heavy, my doctors tell me to avoid it. But if there's no other way at that moment, then I do it.” (Supervisor #2, male)*

Considering work equipment, technical problems like updates in everyday work were added as demanding.

#### Category personal demands

Several personal demands were mentioned within the interviews, including learned behavioural patterns within the supervisors’ career, such as not sparing oneself when working as a service provider, encountering challenges due to prior self-employment or when working as a chef.

*“As I said, I was self-employed for a long time and now I also worked in the gastronomy, but no longer, no longer self-employed, of course you run the store somehow, everything should work or so, but at the end of the evening, yes, you've done your job, you go home, you don't have to go shopping or to the tax advisor and things like that, that is, that was definitely a challenge that you also have to slow down a bit.” (Supervisor #2, male)*

*“Or when the customer says, I need this and so, since I am a service provider through and through, the whole thing is a bit stupid as a service provider sometimes, but it's like that, I actually spare myself quite little, that's unfortunately God's way, I know it too, but, there the leopard can't change its spots.” (Supervisor #15, male)*

Additional demands were posed by the supervisors’ own expectations towards him or herself, supervisors’ own emotions or wanting to help, which is not always possible in an immediate way.

*“Sure also I am a person who has emotions. You can't turn that off. Sometimes I also clatter around.” (Supervisor #11, male)*

*“Then of course you try to get other people involved, to ask and talk to them or something, but if it's something acute, it's sometimes actually more difficult (…), because I'm a person, I just want to help and sometimes it doesn't work right away.” (Supervisor #7, female)*

### Job resources

In addition to the displayed job and personal demands, supervisors also mentioned various resources based on the proposed theoretical framework.

#### Category work tasks

##### Meaning of work

For supervisors, working in social firms was associated with a high meaning of work, e.g. when progress was observed among employees, when they learned new things, had more courage, elevated language and motor skills or when they were no longer afraid of the supervisors absence during vacation. As a result, independence or stability were reported as well as increased working hours or decreased bad days due to the employee’s mental health conditions. Therefore, supervisors tried to find out strengths and weaknesses of the employees and to support them. Overall, supervisors reported higher aspirations of work compared to other settings, that employees were grateful being allowed to work (e.g. after a long period of unemployment) and attributed higher importance to the job, wherefore they enjoyed working with a high motivation.

*“I simply notice that the people in my department are doing well, that they feel good, that they like coming to work, that they are motivated, that they want to get something done, that they want to learn something new. (...) And what it means for these people to have this job. And that is what, yes, what for me is an incredibly good feeling, (…) I am this job. (…) And that's for, for people here, it's an incredible stability in their whole life. And that's fantastic.” (Supervisor #14, male)*

##### Variety of work tasks

Additionally, a high variety of work tasks and a combination of professional and educational tasks, wherefore their motivation was not only accessed via sales numbers only, was stated by supervisors.

*“I think that's good too, that's actually also the profession that I like, it's just a combination of technical work and also pedagogy that I think is good too, yes.” (Supervisor #5, male)*

Moreover, a variety of work tasks was also depicted in terms of the supervisor’s health conditions, including a mixture of sitting activities and such in motion.

##### Work autonomy

The third sub-category refers to the supervisors’ work autonomy and that supervisors could divide up work by himself or herself. Supervisors described despite certain budgetary or staff management guidelines, no control by the management and high levels of trust placed in them.

*“So what I find very nice is that I can, I say, arrange my work myself, that I am alone, so that no one is standing over me and telling me all the time ‘now do this, do this, do this, do this’ and that there is a lot of trust placed in me.” (Supervisor #7, female)*

##### Pleasure in working with people

Overall, supervisors reported pleasure when working with people and finding different ways of communication. For instance, this applied when working with employees holding outsourced jobs from sheltered workshops in social firms.

*“But what I find especially positive is this real feeling of, working in a social profession. Because it really makes a difference how you talk to people and (...) and that's good because we found such a, a, a good way of communication.” (Supervisor #14, male)*

However, supervisors described also that they needed to acclimate when communicating with hearing impaired employees.

#### Category work organisation

##### Participation in shaping the structure of the social firm

Supervisors reported participation in shaping the structures of the social firm in many ways like changes of the supervisors own or the employees’ workplaces, in terms of a further development of processes and workflows, shift or holiday schedules, personnel decisions, the introduction of substitution arrangements or as applied for the gastronomy sector within the menu. Overall, supervisors could ask for support to decrease administrative or maintenance tasks and were able to influence decisions due to their expertise.

*“Yes, if it's just a matter of someone, I'll say now not fitting into the team, then I can decide that. That is, that is also part of my job. Because only I am able to judge that.” (Supervisor #16, male)*

In general, supervisors highlighted a flat hierarchy and communication at eye level in the social firm.

*“That people communicate at eye level, so the hierarchy is very flat here, I think that's very good.” (Supervisor #13, female)*

##### Trainings and seminars

As a basis for working in social frims, trainings and seminars on different topics were offered for supervisors, including occupational health-related topics like prevention and occupational safety, employee or disability-related topics, like sign language for beginners, basic pedagogical skills, interaction with staff, mental health conditions, forensics or other relevant topics like safe driving training or agile management techniques to promote cooperation and organisation or professional trainings. In some cases supervisors were able to reflect and ask questions after four weeks after the seminar. Likewise, there was also the possibility for supervisors to select seminars for employees.

*“Yes, (...) we have really good, good opportunities and good courses we can attend there.” (Supervisor #3, male)*

Individual supervisors rated the seminars as less helpful, though a reflection of a successful cooperation in their social firm was enabled.

##### Meetings on a regular basis

Additionally, meetings for improvements or weekly planning processes were scheduled, e.g. among supervisors, in team meetings in or between different departments and together with all employees. Overall, an open culture of discussion and feedback among supervisors was reported. Organisational aspects included the preparation of a protocol with responsibilities and offers for mutual support as well as the involvement of a social education worker or sign language interpreter.

*“I think so too, so we're really, so super set up and if something's bugging us or something's stressing us out, we either say it immediately, so we immediately go to the next one and say watch out, so this and this didn't go well, this needs to be changed or like I said, we discuss it in our groups, in our round tables.” (Supervisor #4, female)*

Individual supervisors reported that conducting various meetings on a regular basis was considered as time-consuming but useful.

##### Substitution arrangements

Supervisors were able to rely on fixed substitution arrangements in contrast to the situation before, partly after consultation with customers, if those arrangements could cause time delays.

*“But in the meantime everyone has a substitute, as I said, we are constantly working on improvements and we all have a substitute and if the substitute is not there, then we still have someone who can step in again, so I don't have to worry about that. That was always such a problem, you couldn't take a vacation, you went to work sick, because you thought ‘oh God they can't do it’”. (Supervisor #4, female)*

Other supervisors illustrated that substitution arrangements were still being implemented.

##### Cooperation and exchange within the social firm

A cross-departmental exchange was described as a job resource, where supervisors liked to visit different departments to help, keep close contact and get involved in the work (e.g. in sales and customer service) or as applied to the gastronomy sector with a close exchange between service and kitchen departments.

*“Yes, we are always very good at exchanging ideas among ourselves and I think that works really well here in our company, we talk to each other a lot and also across departments quite a lot.” (Supervisor #3, male)*

Overall, individual social firms were described to be relatively small business which couldn’t afford everything and therefore benefited from higher-level structures of their parent company, e.g. through the use of work equipment.

##### Cooperation and networking with external organisations or players

If in smaller social firms the direct supervisor also held the position of the managing director, then cooperation with external organisations was described as beneficial.

*“Otherwise, the cooperation (...) works out very well with the Job Center for the severely disabled, they are highly motivated and support you where they can, that has to be emphasised.” (Supervisor #8, male)*

Also networking with (industry) players was highlighted by supervisors, e.g. with other retailers for technical questions or for information on political structures.

*“Otherwise (...) I am also very well connected with the (...) scene in (name of the city). That's why I can, well, I just have my retailer colleagues there for technical questions.” (Supervisor #11, male)*

##### Working hours

Regarding the working hours of social firm’s supervisors, early closing times and free time on weekends especially in the gastronomy sector were highlighted as job resources. Additionally, the use of flexible working hours without making prior arrangements was underlined as well as the use of working time accounts to reduce overtime.

*“I just go half an hour earlier today or I just come half an hour later or so, I don't have to arrange it beforehand, so there I have an uncanny, yes, yes, uncanny freedom (...) of course I only go if the kitchen is reasonable and, and nothing can go wrong anymore in quotation marks, sure, but I can then estimate that very well and that is uncannily, yes, advantageous.” (Supervisor #10, male)*

To work independent of location only applied for supervisors in appropriate sectors, which results in an elevated compatibility with other activities. Other individual arrangements referred to the reduced number of working hours for supervisor’s relief or a (desired) permanent availability of the supervisor (also during vacation), in order to support employees in their decisions.

#### Category social relations

##### Working atmosphere

Mainly, supervisors mentioned a good working atmosphere in the social firm. Factors like fun among colleagues, a young team of supervisors, affectionate, harmonious and collegial interactions in the team, diversity, a good contact between supervisors and employees despite diverse emotions, employees looking out for each other and private activities among employees contributed to this resource.

*“So positive is actually working with the, with the employees for me. And that from the very beginning. (...) I've been working with some of the people for (...) years. I haven't left, and they're all staying here. We are an incredibly large team and a close-knit team. We also do a lot of private things. (…) Well not me, but the people among themselves. So that's why it's just very, so there is no aggr/ in every kitchen, for example, there is very much, very aggressiveness, very much aggressiveness. Not at all in our kitchen. (…) So of course everyone has to work hard. (…). But it's just a very, very quiet, very peaceful, peaceful atmosphere and, and that's just very pleasant.” (Supervisor #16, male)*

Overall, supervisors described that employees liked to come to work (and were grateful to be allowed to work), as well as supervisors themselves.

##### Social support of colleagues

Beside a good working atmosphere, supervisors reported high levels of social support in the social firm, when asking colleagues for help, e.g. when many employees are on vacation or for customer-related demands. A close exchange among colleagues, who learn from each other was described as well as colleagues who watch out for each other (e.g. concerning breaks). Likewise, supervisors mentioned support for administrative tasks, support from freelancers or for difficult conversations:

*“So, for example, if I have a difficult conversation with an employee, (…) and I don't want to have that conversation, then I go to (name of the colleague) and say, you talk to him, I need a buffer, I don't want to talk to him directly, because I work with him every day, could you do that please.” (Supervisor #4, female)*

A prerequisite for successful support of the supervisor was mentioned to be compatible personnel who is able to deal with employees with disabilities and their needs.

##### Social support of the management

Supervisors experienced social support from management, for instance for communication-related demands in case of problems, inquiries or needs, for administrative tasks, or for pedagogical-related demands (due to qualification of the management), e.g. when dealing with aggressive employees.

*“From the management there is always a lot of appreciation and communication and also offers of help, if something is stressful or something went badly or went well, there is always positive feedback and exchange is always offered, so you have the feeling that you always find open ears with your problems or not problems but requests, needs.” (Supervisor #13, female)*

##### Pedagogical support

Partly, pedagogical support was offered e.g. by trained colleagues or specialists for work and career promotion of sheltered workshop employees, by social education workers or by trainees for guiding the team.

*“We have one, one FABler [specialist for work and career promotion], so to speak, who is our, our contact person when there are difficult issues with, with the people.” (Supervisor #10, male)*

More detailed, materials and tips for team meetings, such as motivation cards, barometers or meetings in standing position were provided by social education workers for individual supervisors.

##### Appreciation

Appreciation for their work with colleagues or the final product, either from their employees, from the management (even though it took some time for individual supervisors to experience appreciation of the management), from the board or shareholders, as well as from supervisors themselves was stated.

*“Yes, I am very satisfied, I feel or experience appreciation here every day which I find absolutely pleasant (...) One thing is simply the togetherness, that everyone here is helpful, regardless of whether it is from the target group or not. There is always a lot of appreciation from the management.” (Supervisor #13, female)*

In case feedback for their work was not provided, it was actively requested from individual supervisors.

##### Customer satisfaction

Furthermore, supervisors in social firms highlighted customer satisfaction as a resource, including nice guests and quick feedback on their work as applied especially for the gastronomy sector.

*“The direct guest contact, I also find very well, that you actually get feedback for what you do (…) that you can assess the results very quickly, that doesn’t apply for many other professions.” (Supervisor #5, male)*

##### Company outings

Company outings in social firms were added as a resource for team building, including e.g. going out to eat or to the cinema, forest tours, cooking, barbecue or game parks.

*“We also had regular get-togethers with everyone. So sometimes we spontaneously brought out the grill and then we had a barbecue. And even if it's only for an hour (...) and of course it's not organised by one person, but by everyone.” (Supervisor #16, male)*

##### Provision of a social psychiatric service

The provision of a social psychiatric service for employees and family members was also described as a job resource by supervisors.

*"What I also find quite well is that we have a social psychiatric service that we can turn to, I think, which is always free of charge.” (Supervisor #7, female)*

#### Category work environment

Considering the results of the interviews with supervisors the provision of an ergonomic work environment was displayed as a job resource, including ergonomic office chairs, hand mats, suitable screen height, extra screens, height adjustable desks, work equipment for lifting or an alternation between sitting and standing activities.

*"Here in the office, everything is done to ensure that we have a good workplace, that we have the right chairs, that we have such mats where the hand is supported (…) or that the screen is at the right height or that it is ergonomic, the workstation, so a lot is done for that, we just have to open our mouths when we need something, (…) for example, I also have a height-adjustable desk, I can also stand up, so actually quite a lot is done to ensure that we are healthy and stay healthy.”(Supervisor #4, female)*

In addition, spacious work environments, green space for walks during breaks or a clear allocation of the premises were mentioned as well. For smaller social firms, the use of work equipment from the parent company also played a role in this context.

#### Category personal resources

Patience and being calm when working with employees with disabilities was stated as a personal resource. This applied to situations where things often had to be explained, questions were asked quite often or when work tasks in progress had to wait for some time. Nevertheless, supervisors tried not to show internal tension to the outside, kept a level of objectivity or tried to withdraw for some time. Overall, individual supervisors added that they had become calmer in the course of time compared to former self-employment.

*“But I’m a very patient person, and then my colleagues always say, ‘You've already explained this to him five times and you're still so calm’- ‘yes, but I know where I work.’ ” (Supervisor #4, female)*

*“Well, I'm just not a person who quickly degenerates into stress, I say, if something arouses, I try to settle everything calmly, even if it sometimes looks different inside me, but outwardly I try not to transfer.” (Supervisor #7, female)*

Other personal resources of supervisors in social firm consisted of empathy in dealing with employees, e.g. due to own experiences with a mental health conditions, optimism or relaxation about own perfectionism:

*“I've never done any major pedagogical training (…). But I would describe myself as quite an empathetic person, and that naturally works to my advantage. So you can deal with the people quite well.” (Supervisor #11, male)*

*“If you work outside in the general labour market, then everything must look just the same as the other and I always say, if that is the case that it is 70 percent of what you want to have, or 50 percent, then it is still good, right, so we do not have to give 100 percent, so we know where we are and the guests also know where they go to eat, right.” (Supervisor #4, female)*

## Discussion

To our knowledge this study was presented as the first one using a qualitative approach to gain insights into working conditions of supervisors working in social firms in Germany, offering employment to a significant amount of employees with different types of disabilities. Supervisors reported several job demands, like guiding work tasks, conflicts between social and economic objectives, a lack of social support, or exposure to heat, heavy lifting or constant standing. In contrast, a high meaning of work, possibilities in shaping the structure of the social firm, social support of colleagues or the management and the provision of an ergonomic work environment were stated as job resources. Referring to the second research question on supervisors’ personal demands, learned behaviour patterns within the supervisors’ career, own expectations or emotions as well as wanting to help were displayed. On the contrary, patience, empathy or relaxation about own perfectionism were presented as resources.

### Social firm specific job demands and resources of supervisors

The main finding of this paper highlights job demands and resources specific for the setting of social firms when employing a significant number of disabled employees. On the one hand concerning their *work tasks*, supervisors reported a high meaning of work and pleasure when working with people. As a result, supervisors shared information about higher levels of independence and stability of employees as well as increased working hours or decreased bad days due to mental illnesses, respectively. Those tendencies were already found in other studies reporting about contributions to employee’s self-confidence, improved work and social skills, well-being, and recovery from mental illnesses (like a reduction in hospital visits since being employed) when working in social firms [2, 3, 10–12, 19, 24, 27, 44]. Apart from advantageous consequences for employees, initial evidence was found in the present study that supervisors also benefit from the progress made by employees and perceiving it as one of the main resource concerning their work tasks.

On the other hand in terms of *work organisation*, different challenges when running a day-to-day business and tensions in balancing social and economic objectives were reported by supervisors. Especially staff scheduling was mentioned as demanding in the present study, when taking absences or emotional crises into account. These results were in line with the current state of research [45] presenting a two-folded challenge in managing both economic and social processes [8, 13, 26]. More detailed, Buhariwala et al. [8] and Wilton et al. [26] displayed tensions when allocating working hours taking merit, seniority, individual preferences as well as operative characteristics into account resulting in higher amounts of administrative work or the adjustment of staffing ratios. Given the fact that social firms serve as companies on the general labour market rather than rehabilitative organisations, Paluch et al. [13] called for implementing cross-sectoral partnerships connecting economic and social parts, since it was described as challenging for social firms to hire skilled supervisors. Further claims of improved social and pedagogical support were added [13] which were also in line with the present interview results, for instance by non-disabled colleagues in the department to provide reliable assistance.

When working as a supervisor in a social firm, a comprehension of mental health conditions appears to be necessary because specific needs of employees occur less visible or multifaceted [16]. Likewise, the results of the study showed high levels of emotional demands. Compared to results of staff working with people with intellectual disabilities, personal burnout was predicted by emotional demands, as well as role and work–privacy conflicts, job insecurity and feedback [46]. Therefore, supervisors should be provided with suitable support and trainings when dealing with employees with mental health conditions to gain insight into disability-related topics, basic pedagogical skills and interaction with staff [4]. To address the aforementioned challenges when communicating with hearing impaired employees, suitable services and professional support should be added when accommodating working conditions for both the employees, and supervisors’ needs [47]. Further possible recommendations in the area of emotion work were presented by Schöllgen and Schulz [48] and included strengthening social support, work autonomy, relief through social sharing as well as breaks. In the same way, a consideration of emotion regulation competencies already during personnel selection was suggested as well as a further improvement through training [48]. The latter suggestion could also counteract the aforementioned conflicts with non-target group employees.

Further job demands in the context of *social relations* emerged for instance with regard to lacking appreciation from the management. When comparing the results to quantitative research from professionals of German sheltered workshops [49], it became evident that there was an association between a lack of appreciation of their work from the management and burnout. Other factors associated with burnout included insufficient predictability of work and high physical demands as well as a low sense of community among professionals [49]. However, results are comparable in a limited way and only resonated partly with our study, as supervisors reported e.g. a good working atmosphere in social firms. Overall, to facilitate the development of social support and working atmosphere further, outings or social events could be suggested in addition to anchoring the principle of mutual support and trust as a guiding principle, trainings and seminars to raise awareness among supervisors to allow and promote support processes and ensuring transparency of decisions making and information flow [50].

Concerning arousing conflicting situations in social firms, supervisors informed about different domains of conflicts: between employees, among supervisors and between supervisors and management. Gaining insight into the first domain of conflicts, it was found in previous research from the general labour market that supervisors’ conflict management behaviour (rated by employees) can have both an intensifying and a buffering effects on the connection between conflicts and conflict-related stress (applying third-party forcing or avoiding behaviour vs. leaders’ third-party problem-solving [51]). On a team level, additional results presented not only the important role of the leader, but also of the team, which can influence their well-being through open conflict norms, which should be created together [52]. Likewise, it was suggested to strengthen leaders as well as the management’s problem-solving behaviour involving each part to present their viewpoints which appears especially important due to varying skills of employees in order to increase ones feelings of control [51].

### Task and sector specific job demands and resources of supervisors in social firms

Moreover, task and sector specific job demands were also identified within the interviews. In general, sectors of social firms were originally focused on the “4F jobs”, including food (gastronomy), filth (cleaning), filling (packaging), and flowers (landscaping/gardening) [53]. On the contrary, results from further studies [8, 22, 23, 26, 54] as well as from our sample underlined a broader spectrum indicating a more heterogenic setting of social firms. For instance, job demands and resources specific for employees in the gastronomy sector were partly in line with those from other companies in the general labour market. A high work intensity, unpredictability as well as job demands due to overtime were described in our results and in the current state of research [55]. The state of working hours at weekends was mentioned as an aggravating factor in the literature [56], which however, was not emphasized in the present study. Supervisors also benefited from the state of working hours without work in the evening hours or on weekends based on the needs of employees.

Further job demands related to the *work environment* in the gastronomy sector were reported and partly reinforced by the interview results, including service-related physical demands like walking and standing as well as carrying heavy dishes and a high noise level [55, 57]. Chefs are also exposed to walking and standing for long periods as well as heavy lifting, carrying, bending forward, and stooping. Likewise, noise, temperature fluctuations, fumes, dust, smoke, wetness and humidity were also highlighted [45, 55]. In the sectors of gardening and landscaping, crafts, cleaning services, laundry and transport, personal protection equipment, occupational safety and ergonomic workplace design were discussed in other survey results from social firms in Germany [45]. Traced back to the heterogeneity of sectors of social firms, further insights on task and sector specifics as well as resulting needs for support are needed.

### General considerations on job demands and resources of supervisors

Other more general topics on work tasks and organisation regardless of the sector were also mentioned within the interviews, including e.g. work autonomy, flexibility or mechanisms for participation. In fact, German survey results from a sheltered workshop and homes for disabled people included three predicting factors associated with emotional exhaustion: latitude in decision-making, workload, and male gender [58]. Systematic reviews that focused on supervisors’ job demands and resources in general came to similar conclusions, highlighting the supervisors’ work autonomy, job security and social support as mental health promoting factors [59]. Therefore, both a horizontal and a vertical scope of action (performing structurally identical tasks vs. tasks with different skills at different levels) should be reflected in a differentiated manner and in relation to competence and qualification issues [60] as well as strategies to reduce emotional exhaustion aiming at the work organisation [58]. With regard to the flexibility of supervisors in terms of work location and hours, it was shown in the past and to some extent also in our study that various models are already offered and used, such as trust-based working hours. These topics appear to be particularly relevant not only concerning employer attractiveness and the recruitment or retention of supervisors, but also concerning the compatibility of work and private life, motivation, productivity and creativity [61]. However, results on flexibility are only transferable to a limited extent with regard to the depicted sectors, work tasks and employees’ needs for support.

On the other hand, the development of a participatory organisational culture in combination with flat hierarchies was depicted. Therefore, guidelines may help to create a framework for responsible actors, since both supervisors and management of social firms were described as not fully equipped to maintain and facilitate participatory structures or demands related to business or production may interfere with such processes [13]. In the same course, rules for digitalization processes could be discussed in teams in a participatory way and afterwards communicated to employees and/or customers for both dealing with flexibility (e.g. regarding the availability outside core hours), and with communication behaviour or media (e.g. defining adequate communication media) [62].

### Supervisors’ personal demands and resources

On a personal level, various resources were named by supervisors, such as patience, optimism, empathy or a relaxed dealing with perfectionism. Results also resonated with findings from related fields, presenting e.g. the ability to maintain inner calmness and remain objective [63] or empathy [64] as main personal resources. In general, personal resources (e.g. like optimism) were partly evaluated as moderating factors when linking environmental resources and work engagement, which is why it could be deduced that environmental resources had an impact on the development of personal resources [65]. As a consequence, the promotion of environmental resources should be considered not only for employees with disabilities in the first place, but also for their supervisors.

On the contrary, personal demands such as own expectations or emotions as well as wanting to help were described in present results. Referring to the latter, past research [66, 67] assumed a link between the compulsion to help and burnout especially in social professions, since own needs and desires may be suppressed combined with low levels of self-esteem and a simultaneous prioritization of the needs of supervised persons. Furthermore, high needs to receive recognition from the management or colleagues in return for a high level of commitment were mentioned. If this appreciation is lacking despite high efforts – as also partly described in the interview results – this can also be a reason for exhaustion [67]. In addition to this, our study revealed further personal demands that received less attention in previous results like different learned behaviour patterns within the supervisors’ career (e.g. after being self-employed). Also against this context, suitable trainings when reflecting own behavioural patterns might be a helpful tool, especially when facing a double burden of social and economic objectives.

### Research implications

The exploratory qualitative study was based on a well-researched theoretical framework (JD-R-Model), which was transferred to the setting of social firms and its supervisors. The results contributed to an increase of knowledge in a little researched field providing various starting points for workplace health promotion interventions. However, the current body of research is characterised by studies analysing the work and health situation of employees of social firms in the first place [7]. Therefore, further research activities should include longitudinal studies with larger sample sizes of supervisors examining health-related outcomes and in turn e.g. turn-over intentions (also in comparison to other companies on the general labour market) when being employed in social firms facing demands resulting from managing both economic and social processes. Results from related fields show several factors directly and indirectly linked to turnover intentions, like work satisfaction, job strain, younger age and more simple subjective labour conditions [68] as well as higher levels of burnout [46].

Sector and task-specific activities should also be taken into account as part of future research, e.g. in gastronomy, industrial or craft services, retail, gardening and landscaping, industrial production or facility management. In general, large-scale studies found that the job of supervisors in general was characterised by various demands such as interruptions at work, strong time pressure, and the simultaneous supervision of diverse tasks (demands increased with the number of employees to be supervised) [56]. However, leaders also had more resources than employees, such as a greater work autonomy or freedom in planning or scheduling. Despite these working conditions, demands were often associated with health impairments [56, 59] (for instance, strong deadline and performance pressure was associated with an increased number of health complaints and intensified with the other demands mentioned [56]). Those combinations of demands were also presented in our exploratory study, wherefore a need for additional research is required. Conducting further research is also crucial concerning the current COVID-19 pandemic and its impacts on supervisors and employees in social firms. The temporary closure and ongoing implementation of short-time work in social firms was reported as stressful, when employees faced e.g. a lack of routine and supervisors had to deal with less staffing and interception of work [69].

Additional research is also needed on the topic of leadership. Not only for maintaining supervisors’ health and productivity-related outcomes, but also to offer sufficient resources for their employees and to strengthen their character as role models considering work- and health-related behaviour [28]. Other research priorities arise on the basis of the company size. Results from the general labour market found that the smaller the company, the greater the decrease in agreements to take care of the employees’ health as a company [70]. Since social firms are often characterised as small and medium-sized companies, it can be assumed that these challenges also apply to working conditions of supervisors, but less - as shown in the current state of research - for its employees [7]. Therefore, further research is also needed concerning the development, implementation and evaluation of workplace health promotion interventions especially for this company size.

### Practical and policy implications

The overview of working conditions of supervisors in social firms underlines the significance of supportive working conditions. Therefore, six main implications can be derived (displayed in Table 3) and classified into behavioural-related implications (by influencing individual behaviour) and structural-related implications (by changing working conditions) [71].

**Table 3:** Practical behavioural- and structural-related implications for supervisors of social firms

Behavioural-related implications	Structural-related implications
(1) To offer trainings fostering the supervisors’ personal resources and stress management techniques (2) To strengthen health-promoting approaches for work organisation and planning processes when balancing social and economic demands (3) To improve the collaboration with the management, other supervisors, employees and customers, conflict management techniques could be reinforced	(1) To strengthen communication structures in teams, e.g. though regular meetings (2) To maintain social support for supervisors by colleagues, the management and pedagogical staff (3) To improve work organisation and environment, including e.g. the supervisors’ work autonomy, participation or substitution arrangements

On the one hand, implications addressing supervisors’ behaviour include three dimensions: To foster *supervisors’ personal resources* trainings could be offered on dealing with mental illnesses and dwindling patience due to the employees’ forgetfulness, frequently asked questions or high numbers of conversations during a work day. In the same vein, a reflection on one’s own expectations and coping strategies could be offered to promote problem-orientation for situations appraised as changeable. Given the challenges with hearing impaired employees, sign language seminars for beginners could also be supplemented to prevent conflicts within the team. Additionally, stress management techniques could be reinforced how to deal with challenging situations and being aware of the body’s stress reactions, especially applying for situations when facing high workloads and time pressure in combination with employees’ absences or emotional crises.

The second implication on the supervisors’ behaviour takes health-promoting *approaches for work organisation and planning processes* into account. Since challenges regarding instructing activities were presented, as well as conflicts between economic and social goals and dealing with work absences of employees, opportunities and limits as a supervisor can be discussed. In this context, strategies could be developed how to deal with work interruptions in case of a high work intensity or ways to relieve the burden on supervisors when intercepting work. Further starting points could be linked to strategies for dealing with constant accessibility, time management or reflection on own breaks.

The third implication on the supervisors’ behaviour addresses *conflict management techniques* for both conflicts between employees and those among supervisors or with the management. Therefore, the supervisors’ problem-solving behaviour could be strengthened and the implementation of open conflict norms for the team facilitated [51, 52].

On the other hand, structural-related implications could be derived based on the interviews. Initially, *communication in teams* could be strengthened e.g. by means of regular meetings with an open feedback culture, with the support of a social education worker or language interpreter if necessary. Furthermore, communication strategies, means and workflows could be defined. Additional rules for dealing with technology might also be helpful, e.g. retrieving e-mails only at certain times, providing a reliable IT-support or maintaining transparent communication structures.

Additionally, *social support for supervisors* could be promoted considering especially pedagogical support through trained colleagues, specialists for work and career promotion or social education workers. Other types of support could address communication and assistance offers in case of problems, inquiries or needs of employees or when coping with administrative tasks. Those type of support appears to be particularly relevant against the background of the described conflicts between social and economic goals. Overall, the support of supervisors with at least one other employee without disabilities was mentioned in the interviews as a possible approach. In the current state of research, the role of social support was reinforced when being involved in emotional labour [48]. Likewise, the importance of appreciation for all persons involved in a social firm could be promoted.

Further implications for *work organisation and environment* could be derived, including e.g. the emphasis of the supervisors’ work autonomy and possibilities for participation. Additionally, the importance of substitution arrangements and relief for supervisors needs to be highlighted, e.g. by means of mentoring structures among employees, buddy systems, or defined substitutes. With regard to the work environment, a culture of supported and shared lifting, e.g. through instructions, could be implemented, as well as the adjustment of work equipment.

### Strengths and limitations

The current study was presented with several strengths. First of all, taking the exploratory character of the study into account, attention was paid to both a heterogeneous age and gender structure as well as to differences in the number of subordinate employees within the sample. Furthermore, different sectors typical for social firms were considered in order to give a broad overview into the topic of leadership in this kind of setting. When presenting the results, several descriptions and direct quotes from the participants were used to increase trustworthiness of qualitative results. During the data analysis process, discussions among the researchers took place to reinforce the transparency and accuracy of results. Considering the explorative nature of the study, another strength followed from the sample size of the study, which were shown to provide sufficient data to approach saturation in previous studies [72]. Overall, the JDR-model posed an appropriate theoretical framework guiding the research questions and allowing empirical comparisons to the current state of research.

Nevertheless, different limitations should be kept in mind when interpreting the results for policy and practice. Due to the qualitative study design and focus to limited sectors, the results do not allow any generalizations as well as transferability of the findings remains open. Therefore, further quantitative studies are needed depicting the impact of the working conditions in social firms on supervisors’ health and work-related outcomes. Moreover, the current COVID-19-pandemic had some impacts on the present study: First, telephone interviews were conducted instead of the originally planned face-to-face interviews. Even though telephone interviews consist of a location-independent synchronous communication with a resulting decrease of certain social clues and lacking contextual insights on the situation of the interviewee, they were evaluated to maintain high quality data [73–75]. An additional potential interviewer bias on interviewees’ responses and tendencies towards social desirability should be reflected as well as a selection bias based on the sampling approach. Second, the time period in which the interviews were conducted should also be highlighted. The summer period in Germany was characterised by lower incidences of SARS-CoV-2 infections and supervisors and employees were able e.g. to open gastronomy businesses according to hygiene and distance regulations. The last interview was conducted on November, 11, with a participant from a social firm which remained open during the second partial lockdown from November, 2 on. Therefore, transferability of the results to other time periods besides the current pandemic situation may be limited. Likewise, transferability is also restricted for target groups in different countries due to different objectives, management techniques, or legal frameworks. Due to time and resource constraints, it was decided not to collect feedback from interviewees on the transcripts and research findings which could have enhanced validity of results and avoided influencing factors like due to the researchers’ knowledge and expectations [37].

## Conclusion

Results of the exploratory interview study indicated both social firm specific job demands and resources as well as task and sector specific ones. On the one hand, supervisors reported a high meaning of work, a variety of work tasks or a good working atmosphere, but were exposed to conflicts when combining social and economic goals or when intercepting absences due to mental health conditions of employees. On the other hand, task and sector specific working conditions were characterised e.g. by a high work intensity, unpredictability as well as job demands due to overtime. In the area of personal resources, results presented patience or empathy as well as demands learned behaviour patterns within the supervisors’ career as central topics for supervisors. Overall, there is a need for further research concerning health-related outcomes of supervisors and the development and evaluation of structural and behavioural interventions for workplace health promotion.

## Supplementary Information

**Additional file 1:** COREQ-Checklist.

## Data Availability

The datasets analysed during the current study are not publicly available due to German national data protection regulations but are available from the corresponding author on reasonable request.

## References

[1] Corbière M, Lecomte T (2009). Vocational services offered to people with severe mental illness. J Ment Health.

[2] Svanberg J, Gumley A, Wilson A (2010). How do social firms contribute to recovery from mental illness? A qualitative study. Clin Psychol Psychother.

[3] Williams A, Fossey E, Harvey C (2012). Social firms: sustainable employment for people with mental illness. Work..

[4] Corbière M, Villotti P, Dewa CS, Sultan-Taïeb H, Fraccaroli F, Zaniboni S, Durand MJ, Lecomte T (2019). Work accommodations in Canadian social firms: supervisors’ and workers’ perspectives. Can J Commun Ment Health.

[5] BIH Bundesarbeitsgemeinschaft der Integrationsämter und Hauptfürsorgestellen (2020). BIH-Jahresbericht 2019/2020, Behinderung & Beruf und soziale Entschädigung.

[6] bag if - Das Netzwerk inklusiver Unternehmen. Inklusionsunternehmen nach Branchen. 2020. https://bag-if.de/integrationsunternehmen-in-zahlen/. Accessed 25 Nov 2021.

[7] Kordsmeyer AC, Lengen JC, Kiepe N, Harth V, Mache S (2020). Working conditions in social firms and health promotion interventions in relation to Employees’ health and work-related outcomes-a scoping review. Int J Environ Res Public Health.

[8] Buhariwala P, Wilton R, Evans J (2015). Social enterprises as enabling workplaces for people with psychiatric disabilities. Disabil Soc.

[9] Corbière M, Zaniboni S, Dewa C, Villotti P, Lecomte T, Sultan-Taïeb H (2019). Work productivity of people with a psychiatric disability working in social firms. Work..

[10] Krupa T, Lagarde M, Carmichael K (2003). Transforming sheltered workshops into affirmative businesses: an outcome evaluation. Psychiatr Rehabil J..

[11] Lanctôt N, Durand M-J, Corbière M (2012). The quality of work life of people with severe mental disorders working in social enterprises: a qualitative study. Qual Life Res.

[12] Milton A, Parsons N, Morant N, Gilbert E, Johnson S, Fisher A, Singh S, Cunliffe D, Marwaha S (2015). The clinical profile of employees with mental health problems working in social firms in the UK. J Ment Health.

[13] Paluch T, Fossey E, Harvey C (2012). Social firms: building cross-sectoral partnerships to create employment opportunity and supportive workplaces for people with mental illness. Work..

[14] Villotti P, Zaniboni S, Corbiere M, Guay S, Fraccaroli F (2018). Reducing perceived stigma: work integration of people with severe mental disorders in Italian social enterprise. Psychiatr Rehabil J.

[15] Villotti P, Corbière M, Zaniboni S, Fraccaroli F (2012). Individual and environmental factors related to job satisfaction in people with severe mental illness employed in social enterprises. Work..

[16] Villotti P, Corbière M, Fossey E, Fraccaroli F, Lecomte T, Harvey C (2017). Work accommodations and natural supports for employees with severe mental illness in social businesses: an international comparison. Community Ment Health J.

[17] Villotti P, Balducci C, Zaniboni S, Corbière M, Fraccaroli F (2013). An analysis of work engagement among workers with mental disorders recently integrated to work. J Career Assess.

[18] Villotti P, Corbière M, Dewa C, Fraccaroli F, Sultan-Taïeb H, Zaniboni S (2017). A serial mediation model of workplace social support on work productivity: the role of self-stigma and job tenure self-efficacy in people with severe mental disorders. Disabil Rehabil.

[19] Williams A, Fossey E, Harvey C (2010). Sustaining employment in a social firm: use of the work environment impact scale v2.0 to explore views of employees with psychiatric disabilities. Br J Occup Ther.

[20] Krupa T, Lysaght R (2016). Perspectives on how social business can engender work identity among people with mental illness. J Policy Prac.

[21] Krupa T, Sabetti J, Lysaght R (2019). How work integration social enterprises impact the stigma of mental illness: negotiating perceptions of legitimacy, value and competence. Soc Enterp J.

[22] Wilton R, Evans J (2016). Social enterprises as spaces of encounter for mental health consumers. Area..

[23] Evans J, Wilton R (2019). Well enough to work? Social Enterprise employment and the geographies of mental health recovery. Ann Am Assoc Geogr.

[24] Elmes AI (2019). Health impacts of a WISE: a longitudinal study. Soc Enterp J.

[25] Secker J, Dass S, Grove BOB (2003). Developing social firms in the UK: a contribution to identifying good practice. Disabil Soc..

[26] Wilton R, Evans J (2018). Accounting for context: social enterprises and meaningful employment for people with mental illness. Work..

[27] Lysaght R, Krupa T (2018). The role of social Enterprise in Creating Work Options for people with intellectual and developmental disabilities. J Dev Disabil.

[28] Pundt F, Felfe J (2017). HoL - Health oriented Leadership, Instrument zur Erfassung gesundheitsförderlicher Führung.

[29] Parr AD, Hunter ST (2014). Enhancing work outcomes of employees with autism spectrum disorder through leadership: leadership for employees with autism spectrum disorder. Autism..

[30] Demerouti E, Bakker AB, Nachreiner F, Schaufeli WB (2001). The job demands-resources model of burnout. J Appl Psychol.

[31] Bakker AB, Demerouti E (2007). The job demands-resources model: state of the art. J Manag Psychol.

[32] Demerouti E, Bakker AB (2011). The Job Demands–Resources model: Challenges for future research. SA J Industrial Psychol.

[33] DIN Deutsches Institut für Normung e. V. DIN EN ISO 10075 Ergonomische Grundlagen bezüglich psychischer Arbeitsbelastung. Berlin: Beuth-Verlag; 2018.

[34] Gemeinsame Deutsche Arbeitsschutzstrategie (GDA) (2017). Empfehlungen zur Umsetzung der Gefährdungsbeurteilung psychischer Belastungen.

[35] Bamberg E, Keller M, Wohlert C, Zeh A (2006). BGW-Stresskonzept Das arbeitspsychologische Stressmodell.

[36] Bamberg E, Busch C, Ducki A, Glaveris A (2003). Stress- und Ressourcenmanagement: Strategien und Methoden für die neue Arbeitswelt.

[37] Tong A, Sainsbury P, Craig J (2007). Consolidated criteria for reporting qualitative research (COREQ): a 32-item checklist for interviews and focus groups. J Healthc Qual.

[38] Witzel A (2000). Das problemzentrierte Interview Forum Qualitative Sozialforschung / Forum. Qual Soc Res.

[39] Helfferich C (2005). Die Qualität qualitativer Daten: Manual für die Durchführung qualitativer Interviews.

[40] Kuckartz U (2016). Qualitative Inhaltsanalyse. Methoden, Praxis, Computerunterstützung.

[41] Kuckartz U (2010). Einführung in die computergestützte Analyse qualitativer Daten.

[42] Mayring P (2015). Qualitative Inhaltsanalyse - Grundlagen und Techniken.

[43] Eurostat European Commission (2008). NACE Rev. 2 - Statistical classification of economic activities in the European Community.

[44] Dewa CS, Hoch JS, Corbière M, Villotti P, Trojanowski L, Sultan-Taïeb H, Zaniboni S, Fraccaroli F (2019). A comparison of healthcare use and costs for workers with psychiatric disabilities employed in social enterprises versus those who are not employed and seeking work. Community Ment Health J.

[45] Berufsgenossenschaft für Gesundheitsdienst und Wohlfahrtspflege (BGW) (2019). Erschließung des Kundenbedarfs der Inklusionsbetriebe Ergebnisse einer Kundenbefragung (unpublished, internal report).

[46] Kozak A, Kersten M, Schillmoller Z, Nienhaus A (2013). Psychosocial work-related predictors and consequences of personal burnout among staff working with people with intellectual disabilities. Res Dev Disabil.

[47] Svinndal EV, Jensen C, Rise MB (2020). Employees with hearing impairment. A qualitative study exploring managers’ experiences. Disabil Rehabil.

[48] Schöllgen I, Schulz A (2016). Psychische Gesundheit in der Arbeitswelt - Emotionsarbeit.

[49] Schwangler J, Wahl L, Neuperdt L, Rathmann K (2020). Berufliche Belastungen und Burnout-Risiko von Leitungs- und Fachkräften in Werkstätten für Menschen mit Behinderung: Ergebnisse der bundesweiten WeCareOnline-Studie. Prävention und Gesundheitsförderung.

[50] Stadler P, Spieß E (2012). Mit Verstand und Verständnis - Mitarbeiterorientiertes Führen und soziale Unterstützung am Arbeitsplatz.

[51] Römer M, Rispens S, Giebels E, Euwema MC (2012). A helping hand? The moderating role of Leaders’ conflict management behavior on the conflict–stress relationship of employees. Negot J.

[52] Robijn W, Euwema MC, Schaufeli WB, Deprez J (2020). Leaders, teams and work engagement: a basic needs perspective. Career Dev Int.

[53] Kirsh B, Krupa T, Cockburn L, Gewurtz R (2006). Work initiatives for persons with severe mental illnesses in Canada: a decade of development. Can J Commun Ment Health..

[54] Gilbert E, Marwaha S, Milton A, Johnson S, Morant N, Parsons N, Fisher A, Singh S, Cunliffe D (2013). Social firms as a means of vocational recovery for people with mental illness: a UK survey. BMC Health Serv Res.

[55] Krüger F, Guhlemann K, Beerheide E, Georg A, Goedicke A, Nordbrock C, Seiler K (2018). Arbeit und Arbeitsbedingungen im Gastgewerbe. Gesundheitsgerechte Dienstleistungsarbeit: Diskontinuierliche Erwerbsverläufe als Herausforderung für Arbeitsgestaltung und Kompetenzentwicklung im Gastgewerbe.

[56] Lohmann-Haislah A (2012). Psychische Anforderungen, Ressourcen und Befinden.

[57] Klein Hesselink J, Houtman I, van den Berg R, van den Bossche S, van den Heuvel F (2004). TNO work and employment. EU hotel and restaurant sector: work and employment conditions.

[58] Kowalski C, Driller E, Ernstmann N, Alich S, Karbach U, Ommen O, Schulz-Nieswandt F, Pfaff H (2010). Associations between emotional exhaustion, social capital, workload, and latitude in decision-making among professionals working with people with disabilities. Res Dev Disabil.

[59] Zimber A, Hentrich S, Bockhoff K, Wissing C, Petermann F (2015). Wie stark sind Führungskräfte psychisch gefährdet?. Z Gesundheitspsychol.

[60] Rosen (2016). Psychische Gesundheit in der Arbeitswelt Handlungs- und Entscheidungsspielraum, Aufgabenvariabilität.

[61] Gärtner M, Garten T, Huesmann M (2016). Flexible Arbeitsmodelle für Führungskräfte. Zum Stand der Forschung. Z Arb Wiss.

[62] Böhm S, Baumgärtner MK, Breier C, Götz TM, Walther MD (2019). Gesundheitliche Effekte des digitalen Wandels am Arbeitsplatz - Ergebnisse einer repräsentativen Längsschnittanalyse der Universität St. Gallen im Auftrag der BARMER Krankenkasse.

[63] Poulsen I (2008). Burnoutprävention im Berufsfeld Soziale Arbeit: Perspektiven zur Selbstfürsorge von Fachkräften.

[64] Whitley R, Kostick KM, Bush PW (2010). Desirable characteristics and competencies of supported employment specialists: an empirically-grounded framework. Admin Pol Ment Health.

[65] Xanthopoulou D, Bakker AB, Demerouti E, Schaufeli WB (2007). The role of personal resources in the job demands-resources model. Int J Stress Manag.

[66] Schmidbauer W (2002). Helfersyndrom und Burnout-Gefahr.

[67] Elsässer J, Sauer KE (2013). Burnout in sozialen Berufen - Öffentliche Wahrnehmung, persönliche Betroffenheit, professioneller Umgang.

[68] Hatton C, Emerson E, Rivers M, Mason H, Swarbick R, Mason L (2001). Factors associated with intended staff turnover and job search behaviour in services for people with intellectual disability. J Intellect Disabil Res.

[69] Kordsmeyer A-C, Efimov I, Lengen JC, Harth V, Mache S (2021). “One of My Basic Necessities of Life Is Work. That’s Just Broken Away.”—Explorative Triangulation of Personal and Work-Related Impacts for Supervisors and Disabled Employees in German Social Firms during the COVID-19 Pandemic. Int J Environ Res Public Health.

[70] Pauls N, Pangert B, Lück B (2015). Arbeit und Gesundheit im Wandel - Auswertungen der vier Wellen des iga. Barometers für die Jahre 2004 bis 2013.

[71] Ulich E, Wülser M (2018). Gesundheitsmanagement in Unternehmen - Arbeitspsychologische Perspektiven.

[72] Guest G, Bunce A, Johnson L (2006). How many interviews are enough? An experiment with data saturation and variability. Field Methods.

[73] Novick G (2008). Is there a bias against telephone interviews in qualitative research?. Res Nurs Health.

[74] Opdenakker R (2006). Advantages and Disadvantages of Four Interview Techniques in Qualitative Research. Forum Qualitative Sozialforschung / Forum. Qual Soc Res.

[75] Block ES, Erskine L (2012). Interviewing by telephone: specific considerations, opportunities, and challenges. Int J Qual Methods.

